# LncRNA *SNHG12* promotes cell growth and inhibits cell apoptosis in colorectal cancer cells

**DOI:** 10.1590/1414-431X20176079

**Published:** 2017-02-20

**Authors:** J.Z. Wang, C.L. Xu, H. Wu, S.J. Shen

**Affiliations:** Department of Gastroenterology, The Second Affiliated Hospital of Wenzhou Medical University, Wenzhou, China

**Keywords:** SNHG12, Cell growth, biomarker, Cell apoptosis, Colorectal cancer

## Abstract

Several long non-coding RNA (lncRNA) might be correlated with the prognosis of colorectal cancer (CRC) and serve as a diagnostic and prognostic biomarker. However, the exact expression pattern of small nucleolar RNA host gene 12 (*SNHG12*) in colorectal cancer and its clinical significance remains unclear. The level of *SNHG12* was detected by qRT-PCR in CRC tissues and CRC cells. MTT assay and colony formation assay were performed to examine the cell proliferation of CRC cells transfected with pcDNA-*SNHG12* or si-*SNHG12*. Flow cytometry technology was used to detect cell cycle and cell apoptosis of CRC cells transfected with pcDNA-*SNHG12* or si-*SNHG12*. The protein level of cell cycle progression-related molecules, including cyclin-dependent kinases (CDK4, CDK6), cyclin D1 (CCND1) and cell apoptosis-related molecule caspase 3 was detected by western blot. The effect of *SNHG12* knockdown was examined *in vivo*. Increased levels of *SNHG12* were observed in CRC tissues and in CRC cells. *SNHG12* promoted the cell proliferation of CRC cells. In addition, *SNHG12* overexpression boosted the cell cycle progression of SW480 cells transfected with pcDNA-*SNHG12* and *SNHG12* knockdown inhibited the cell cycle progression of HT29 cells transfected with si-*SNHG12*. *SNHG12* also inhibited the cell apoptosis of CRC cells. We also found that *SNHG12* increased the expression of cell cycle-related proteins and suppressed the expression of caspase 3. Our results suggest that *SNHG12* promoted cell growth and inhibited cell apoptosis in CRC cells, indicating that *SNHG12* might be a useful biomarker for colorectal cancer.

## Introduction

Colorectal cancer (CRC) is the third most common malignant tumor globally (1,2). The high incidence of CRC and CRC-related deaths has been a great threat to public health. In spite of the improvement of diagnosis and treatment of CRC, it remains a severe disease. Progression of CRC is a multi-step process involving the deregulation of several oncogenes and tumor suppressor gene, which might be used as diagnostic and therapeutic targets (3). However, the precise mechanisms of these genes in CRC are poorly understood and novel diagnostic and prognostic biomarkers need to be discovered.

With the advances in next generation sequencing technologies, long non-coding RNAs (lncRNAs) were identified as a new class of non-coding RNA. LncRNA is longer than 200 nucleotides with no protein-coding capacity (4). Mounting evidence suggests that aberrant lncRNA expression participate in a variety of biological processes, such as embryogenesis, stem cell pluripotency, cell growth and the occurrence of malignancies, through regulating gene expression at several levels (5,6). Notably, lncRNA might be correlated with patients' prognosis and serve as a diagnostic and prognostic biomarker for disease. In CRC, various lncRNAs have been identified to be abnormally expressed and related to disease progression (7). For example, Niu et al. (8) found that lncRNA *AK027294* was strongly expressed in CRC and closely correlated with cell proliferation, migration, and apoptosis. *TUG1* was found to indicate a poor prognosis for CRC and promote metastasis by regulating epithelial-mesenchymal transition (9). In addition, Xie et al. reviewed the CRC-associated lncRNAs published recently, including *CCAT1*, *H19*, *HOTAIR*, *UCA1* and *PTENP1* (10). However, no robust tumor markers have been yet identified.

Long non-coding RNA small nucleolar RNA host gene 12 (*SNHG12*) was a novel lncRNA identified to be up-regulated in several cancer cells, such as human osteosarcoma cell, nasopharyngeal carcinoma cell, and human endometrial carcinoma (11–13). Moreover, *SNHG12* played important roles in cancer cell proliferation and migration. However, the exact expression pattern of *SNHG12* in CRC and its clinical significance remains unclear. In the present research, we discovered that *SNHG12* was up-regulated in CRC tissues and cells for the first time. We further detected the effect of *SNHG12* on cell proliferation, cell cycle, apoptosis and the related proteins expression in CRC cells.

## Material and Methods

### Patients and specimens

Human primary CRC tissues and their paired adjacent tissue were obtained from 60 patients at the Second Affiliated Hospital, Wenzhou Medical University. These patients did not receive local or systemic treatment before the operation. All of the tissues were stored at –80°C. An experienced pathologist assessed the differentiation grade, pathological stage, grade and nodal status. All subjects submitted the written informed consent. The study protocol was approved by the Ethics Committee of the Second Affiliated Hospital of Wenzhou Medical University.

### Cell culture and transfection

All human colonic cancer cell lines including SW480, LOVO, HCT116, HT29 and the human colonic epithelial cells HCoEpiC were obtained from the American Type Culture Collection. Cells were cultured in RPMI-1640 supplemented with 10% fetal bovine serum at 37°C in a 5% CO_2_ incubator.

The *SNHG12* expression vector, pcDNA-*SNHG12*, was synthetized and constructed by Ribobio (China). siRNA-*SNHG12* (si-*SNHG12*) which targets *SNHG12* was obtained from Sigma-Aldrich (USA). Cells were transfected with pcDNA-*SNHG12* or siRNAs using Lipofectamine2000 (Life Technologies, USA) following the manufacturer's instructions.

### Quantitative real-time PCR

Total RNA was extracted from tumor tissue samples or cultured cells using Trizol reagent (Invitrogen Inc., USA). Two micrograms of total RNA was reverse transcribed to obtain cDNA using Moloney Murine Leukemia Virus Reverse Transcriptase (M-MLVRT; Promega, USA). Quantitative real-time PCR was performed with 1 μL of cDNA using SYBR green real-time Master Mix (Takara, Japan) on Applied Biosystems 7500 Sequence Detection system (ABI, USA). Glyceraldehyde 3-phosphate dehydrogenase (GAPDH) was used as an internal control to normalize the data. The primers for *SNHG12* and *GAPDH* were as followed: for *SNHG12*, (forward) 5′-TCTGGTGATCGAGGACTTCC-3′, and (reverse) 5′-ACCTCCTCAGTATCACACACT-3′; for *GAPDH*, (forward) 5′-ACACCCACTCCTCCACCTTT-3′ and (reverse) 5′-TTACTCCTTGGAGGCCATGT-3′. Real time PCR was performed in triplicate, and the relative expression of *SNHG12* was calculated using 2^-ΔΔCT^ method.

### Western blot analysis

Total proteins were extracted from cells and protein concentrations were determined using the BCA Protein Assay kit (Takara). Proteins were separated on 12% sodium lauryl sulfate-polyacrylamide gels (SDS-PAGE) and transferred to polyvinylidene difluoride membranes (PVDF; Millipore, USA). After blocked with 5% non-fat skimmed milk powder at 37°C for 2 h, the membranes were incubated with primary antibodies: anti-cyclin-dependent kinase 4 (anti-CDK4) antibody (1:5000, Abcam, UK), anti-CDK6 antibody (1:5000, Abcam), anti-CCND1 antibody (1:5000, Abcam), anti-Caspase 3 antibody (1:5000, Abcam), anti-p-AKT antibody (1:500, Abcam) and GAPDH diluted at 1:2000 (Abcam) for 1 h at 37°C. The second antibody was anti-rabbit IgG-horseradish peroxidase (HRP, 1:4000; Santa Cruz, USA). Proteins were detected by enhanced chemiluminescence as described by the manufacturer (Beyotime, China).

### MTT assay and soft agar colony formation assay

The 3-[4,5-dimethylthiazol-2-yl]-2,5-diphenyltetrazolium bromide (MTT) assay was carried out to detect the cell viability of SW480 cells with pcDNA-*SNHG12* or HT29 cells with si-*SNHG12* at 0, 12, 24, 36, 48, 60 and 72 h of the transfection. The transfected CRC cells (2×10^4^ cells) were seeded on 6-well plates and were washed with PBS, then incubated in MTT solution (5 mg/mL, 100 μL; Invitrogen Inc., USA) for 3 h. After 3 h, 100 μL of solubilization buffer was added to each well. The absorbance of samples at 450 nm was measured using the Thermo Plate microplate reader (Rayto Life and Analytical Science Co. Ltd., Germany).

For the colony formation assay, 800–1500 cells were placed in a 6-well plate and maintained in complete culture medium containing 0.3% agar layered on top of 0.6% agar at 37°C in the presence of 5% CO_2_ for 16 days. We evaluated the colonies containing at least 50 cells. The data of five randomly scored fields were used for statistics.

### Flow cytometry technology to detect cell cycle and cell apoptosis

For the detection of cell cycle, SW480 cells with pcDNA-*SNHG12* or HT29 cells with si-*SNHG12* were harvested after 48 h of transfection. Propidium oxide was used to stain cells with the BD Cycletest Plus DNA Reagent Kit (BD Biosciences, USA). The quantitation of cell cycle distribution was performed with FACScan cytometry (Becton Dickinson, USA). The percentage of the cells in G0-G1, S, and G2-M phases were counted and compared.

An Annexin V-fluorescein isothiocyanate propidium iodide (FITC/PI) apoptosis detection kit (Life Technologies, USA) was used to detect cell apoptosis. SW480 cells with pcDNA-*SNHG12* or HT29 cells with si-*SNHG12* were harvested after 48 h of transfection and washed twice with 1× PBS. Cells were incubated with Annexin-V and PI at room temperature for 15 min in the dark. Then, samples were analyzed by a FACScan (Becton-Dickinson) flow cytometry. Signals Annexin-V−/PI−, Annexin-V+/PI−, and Annexin-V+/PI+ indicated living, early, and late apoptotic cells, respectively. Each experiment was performed in triplicate, and data are reported as means±SD.

### Animal experiments

All animal experiments were performed according to the guidelines approved by the China Association of Laboratory Animal Care. Twelve female 6-week-old BALB/c nude mice were obtained from Vital River Co. Ltd. (China) and maintained under specific pathogen-free conditions. HT29 cells were transfected si-NC or si-*SNHG12* and then injected subcutaneously into the hind limb of BALB/c nude mice (5×10^6^ cells/mouse; n=6 for each group). The tumor size was measured every 7 days for a total period of 28 days and estimated using the equation length×(width)^2^×0.5. The weight of the tumor on the 28th day was measured.

### Statistical analysis

All statistical analyses were performed using SPSS 20.0 software (SPSS, USA). The measured parameters are reported as means±SD. The Fisher's exact test was used to compare categorical data and the Kruskal-Wallis method was used to analyze continuous data. The differences between groups were estimated by the Student's *t*-test. A P value of <0.05 was considered to be statistically significant.

## Results

### *SNHG12* was upregulated in CRC tissues and cell lines

**Figure 1:**
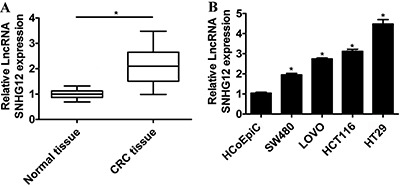
Relative expression of small nucleolar RNA host gene 12 (*SNHG12*) in colorectal cancer (CRC) tissues or normal tissues (*A*) and in different CRC cell lines or the control cells HCoEpiC (*B*) detected by qRT-PCR. Data are reported as means±SD. *P<0.01 *vs* control (Student's *t*-test).

The level of *SNHG12* in CRC tissues and the adjacent normal tissues were detected by qRT-PCR. As shown in Figure 1A, higher level of *SNHG12* was observed in CRC tissues compared to that in normal tissues. qRT-PCR was also conducted to determine the level of *SNHG12* in CRC cells (SW480, LOVO, HCT116, HT29) and the control cells HCoEpiC. *SNHG12* was significantly up-regulated in these CRC cells compared to that in the control (Figure 1B). In addition, the highest level of *SNHG12* was found in HT29 cells and SW480 cells expressed the lowest *SNHG12*.

**Figure 2:**
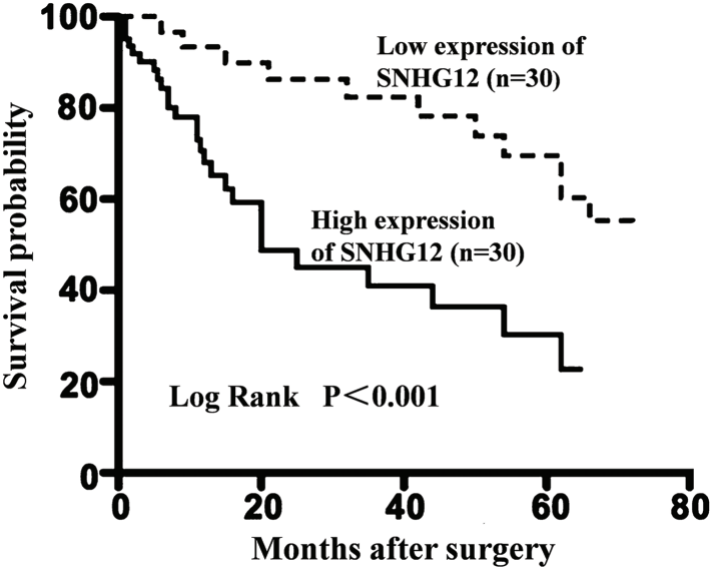
Prognostic significance of high and low expression of small nucleolar RNA host gene 12 (*SNHG12*) in colorectal cancer patients.

To detect the clinical significance of *SNHG12* expression in CRC, 60 patients were divided into *SNHG12* high expression group (n=30) and *SNHG12* low expression group (n=30) according to the cutoff value, which was defined as the median of the cohort. As demonstrated in Table 1, high expression of *SNHG12* in CRC patients was significantly correlated with advanced tumor stage (P=0.007) and large tumor size (P=0.004). In addition, tumors with high expression of *SNHG12* were associated with worse overall survival in CRC patients (P<0.001; Figure 2). The 5-year survival rate of *SNHG12* high expression group reached 33.3% and the rate in low expression group was 70%. These data indicated that *SNHG12* may act as a potent biomarker for predicting prognosis in CRC patients.

**Table:**
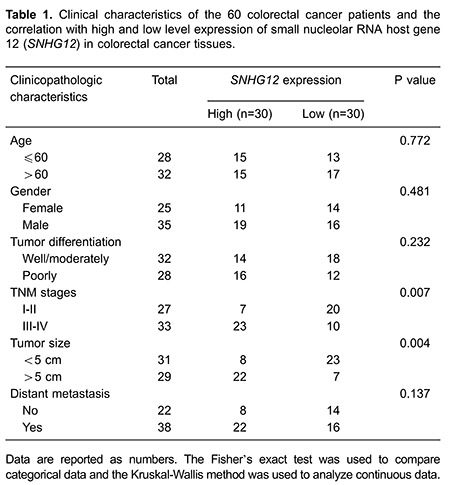

### *SNHG12* promoted proliferation of CRC cells

**Figure 3:**
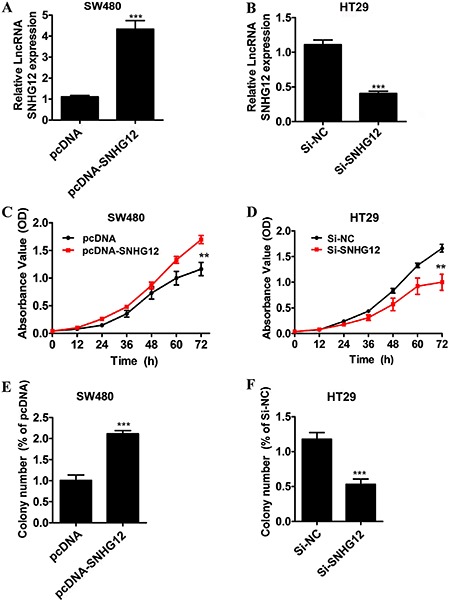
Effect of small nucleolar RNA host gene 12 (*SNHG12*) on proliferation of colorectal cancer cells. The expression of *SNHG12* was detected by qRT-PCR in SW480 cells transfected with pcDNA-*SNHG12* (*A*) and in HT29 cells transfected with si-*SNHG12* (*B*). MTT assay was performed to determine the proliferation of SW480 cells transfected with pcDNA-*SNHG12* (*C*) and HT29 cells transfected with si-*SNHG12* (*D*). Colony formation of SW480 cells transfected with pcDNA-*SNHG12* (*E*) and HT29 cells transfected with si-*SNHG12* (*F*). Data are reported as means±SD. **P<0.05 *vs* the control (Student’s *t*-test); ***P<0.01 *vs* control (Student’s *t*-test).

To investigate the function of *SNHG12* on the cell proliferation of CRC cells, SW480 cells or HT29 cells were transfected with pcDNA-*SNHG12* or si-*SNHG12*, respectively. The result of qRT-PCR showed that the expression of *SNHG12* was greatly up-regulated in SW480 cells transfected with pcDNA-*SNHG12* and down-regulated in HT29 cells transfected with si-*SNHG12* (Figure 3A and B). Cell viability of pretreated SW480 cells and HT29 cells were detected by MTT assay. The results showed that overexpression of *SNHG12* significantly promoted the cell viability of SW480 cells and *SNHG12* knockdown inhibited the cell viability of HT29 cells (Figure 3C and D). The result of colony formation assays indicated that colony formation was significantly promoted by *SNHG12* overexpression and remarkably inhibited by *SNHG12* knockdown (Figure 3E and F). These data indicated that *SNHG12* promoted the proliferation of colorectal cancer cells.

### *SNHG12* promoted cell cycle of CRC cells

**Figure 4:**
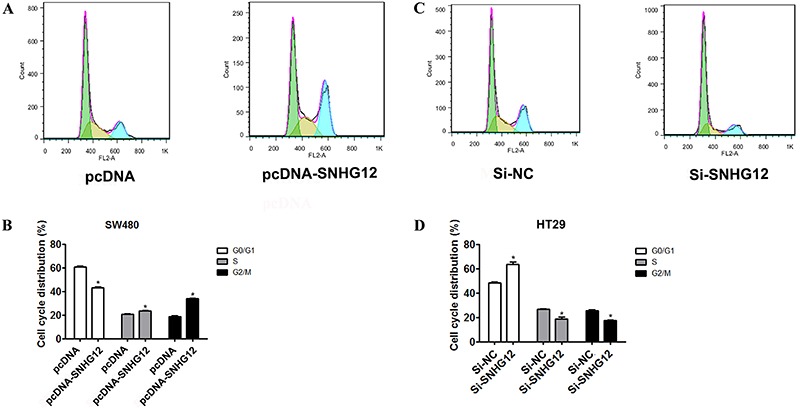
Flow cytometric analysis for cell cycle progression (*A*) and the corresponding statistical result (*B*) in SW480 cells transfected with pcDNA-*SNHG12* and in HT29 cells transfected with si-*SNHG12* (*C* and *D*, respectively). Data are reported as means±SD. *P<0.01 *vs* control (Student’s *t*-test).

To explore the effect of *SNHG12* on the cell cycle of CRC cells, flow cytometric analysis was performed. As shown in Figure 4A and B, *SNHG12* overexpression significantly reduced the number of SW480 cells in the G0/G1 phase and increased the number of SW480 cells in the S phase, which suggested that the cell cycle progression of SW480 cells transfected with pcDNA-*SNHG12* was promoted. On the other hand, *SNHG12* knockdown inhibited the cell cycle progression of HT29 cells transfected with si-*SNHG12* (Figure 4C and D).

### *SNHG12* inhibited apoptosis of CRC cells

**Figure 5:**
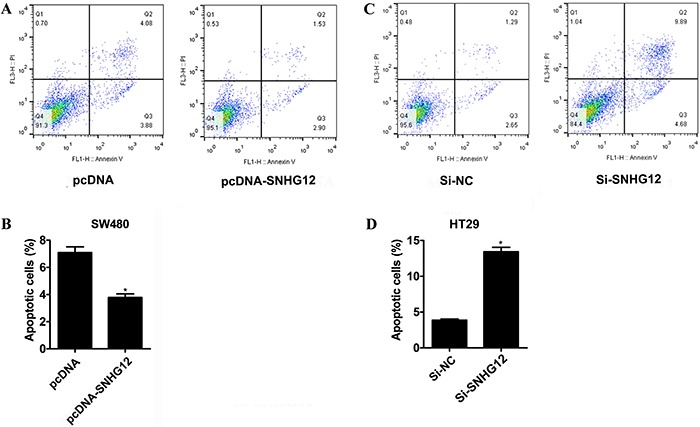
Flow cytometric analysis for cell apoptosis (*A*) and the corresponding statistical result (*B*) in SW480 cells transfected with pcDNA-*SNHG12* and in HT29 cells transfected with si-*SNHG12* (*C* and *D*, respectively). Data are reported as means±SD. *P<0.01 *vs* control (Student’s *t*-test).

To investigate the effect of *SNHG12* on the cell apoptosis of CRC cells, SW480 cells were transfected with pcDNA-*SNHG12* or pcDNA for 48 h, then stained with annexin V and propidium iodide (PI), followed by detection using flow cytometry. The results showed that *SNHG12* overexpression can obviously suppress cell apoptosis of SW480 cells (Figure 5A and B). In addition, *SNHG12* knockdown induced cell apoptosis of HT29 cells transfected with si-*SNHG12* (Figure 5C and D).

### *SNHG12* promoted the expression of cell cycle-related proteins and suppressed the expression of caspase 3

**Figure 6:**
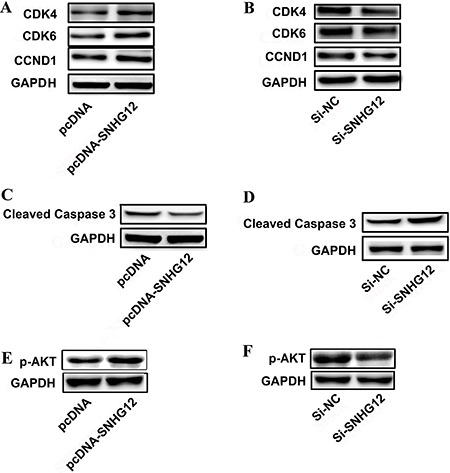
Protein level of CDK4, CDK6, CCND1 (*A)* and of caspase 3 (*C*) in SW480 cells transfected with pcDNA-*SNHG12*. Protein level of CDK4, CDK6, CCND1 *(B)* and of caspase 3 (*D*) in HT29 cells transfected with si-*SNHG12*. Protein level of p-AKT in SW480 cells transfected with pcDNA-*SNHG12 (E)* and in HT29 cells transfected with si-*SNHG12* (*F*).

To explore the molecular mechanisms by which *SNHG12* contributes to cell cycle and cell apoptosis of CRC cells, we performed western blot to detect the protein level of cell cycle progression-related molecules, including CDK4, CDK6), cyclin D1 (CCND1) and cell apoptosis-related molecule caspase 3. CDK4, CDK6 and CCND1 were found to be up-regulated in SW480 cells transfected with pcDNA-*SNHG12* (Figure 6A) and down-regulated in HT29 cells transfected with si-*SNHG12* (Figure 6C). The result of western blot showed that lower protein level of caspase 3 was observed in SW480 cells transfected with pcDNA-*SNHG12* (Figure 6B). *SNHG12* knockdown enhanced the protein level of caspase 3 in HT29 cells (Figure 6D). As it is well known that PI3K/AKT signaling pathway plays vital roles in regulating cell proliferation, cycle and apoptosis, we asked whether *SNHG12* could also regulate PI3K/AKT signaling pathway. As shown in Figure 6E and F, overexpression of *SNHG12* significantly promoted the expression of phosphorylated AKT, while *SNHG12* knockdown inhibited the expression of phosphorylated AKT.

### *SNHG12* knockdown inhibited tumor growth *in vivo*

**Figure 7:**
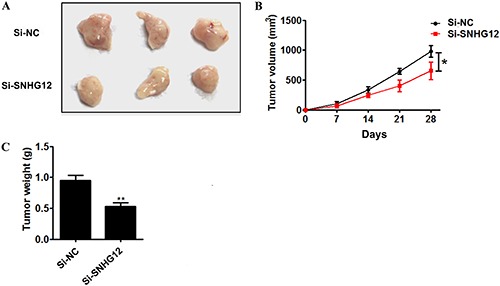
Effect of *SNHG12* knockdown on colorectal cancer tumor growth *in vivo*. The subcutaneous xenotransplanted tumor model of HT29 cells in nude mice was established. HT29 cells were transfected with si-NC (n=6) or si-*SNHG12* (n=6). *A*, tumors in mice inoculated with HT29 cells treated with si-NC or si-*SNHG12*. *B*, tumor size in the two groups. *C*, tumor weight in the two groups. Data are reported as means±SD. *P=0.05, **P<0.01 *vs* control (Student’s *t*-test).

To detect the effect of *SNHG12* knockdown *in vivo*, a nude mouse xenograft model of HT29 cells was established. HT29 cells were transfected with si-NC or si-*SNHG12* and subcutaneously inoculated into the nude mice (n=6 for each group). As shown in Figure 7A and B, *SNHG12* knockdown inhibited tumor growth significantly after inoculation for 28 days. In addition, the tumor weight on 28th day in mice inoculated with HT29 cells transfected with si-*SNHG12* was significantly smaller than that of the controls (Figure 7C). Results *in vivo* indicated that *SNHG12* knockdown inhibited tumor growth.

## Discussion

In this study, we observed increased levels of *SNHG12* in CRC tissues and in CRC cells including SW480, LOVO, HCT116, HT29. High expression of *SNHG12* in CRC patients was significantly correlated with advanced tumor stage and large tumor size. In addition, tumors with high expression of *SNHG12* were associated with worse overall survival in CRC patients. The results of MTT assay and colony formation assay indicated that *SNHG12* promoted proliferation of CRC cells. In addition, *SNHG12* overexpression boosted cell cycle progression of SW480 cells transfected with pcDNA-*SNHG12* and *SNHG12* knockdown inhibited the cell cycle progression of HT29 cells transfected with si-*SNHG12*. *SNHG12* also inhibited cell apoptosis of CRC. We further found that *SNHG12* increased the expression of cell cycle-related proteins and suppressed the expression of caspase 3. *SNHG12* knockdown inhibited the tumor growth *in vivo*. Taken together, our results suggest that *SNHG12* promoted cell growth and inhibited cell apoptosis in CRC cells and might be a potent biomarker for predicting prognosis in CRC patients.

To date, accumulating evidence has suggested that the dysfunctional activities of lncRNAs might be associated with tumor initiation and progression of CRC, which further promotes the application of lncRNAs as cancer diagnostic or prognostic biomarkers (14,15). Yuan et al. (16) demonstrated that lncRNA-*CTD903* was an independent predictive factor of favorable prognosis for CRC and acted as a tumor suppressor to inhibit cell invasion and migration. In addition, the extensively studied lncRNA *MALAT-1* was also expressed at a high level in CRC tissues (17). Apart from the abnormal expression of lncRNA, some genetic variants of lncRNA are also correlated with the risk of CRC. For example, rs2839689 in lncRNA *H19* was reported to contribute to the susceptibility to CRC in a Chinese population study by Li et al. (18).

In this study, we found that a novel lncRNA *SNHG12* was up-regulated in CRC tissues and cells. *SNHG12* was also related to the prognosis of CRC patients. Ruan et al. reported that *SNHG12* contributed to cell proliferation and migration by upregulating the expression of angiomotin in human osteosarcoma cells (12). According to Peri et al. (19), *SNHG12* was confirmed to be differentially expressed in nulliparous and parous breast tissues. In low-passage human nasopharyngeal carcinoma HNE2 cells, the results of lncRNA microarray analysis and qRT-PCR confirmed that *SNHG12* was overexpressed following TP53 overexpression.

To further investigate the mechanism by which *SNHG12* contributed to cellular proliferation and apoptosis of CRC cells, we explored the expression of CDK4, CDK6, CCND1 and caspase 3. In SW480 cells transfected with pcDNA-*SNHG12*, CDK4, CDK6 and CCND1 were found to be up-regulated and Caspase 3 was down-regulated. It is known that cyclins, CDKs, and cyclin-dependent kinase inhibitors play important roles in the functioning mechanism of the cell cycle (20). The activation of CDK4 or CDK6 is required for cell cycle G1/S transition. CDK4 or CDK6 can interact with tumor suppressor protein Rb. In cancer cells, CCND1 is upregulated and activates CDK4/6, whereas Rb is deactivated, resulting in the dysregulation of the DNA-damage repair system and acceleration of the cell cycle (21). Caspase 3 is the most important of the "executioner caspases" in the process of apoptosis, which could begin to disassemble the cell by activating DNA degrading enzymes and degrading the cellular architecture (22). Previous studies have shown that some lncRNAs could regulate cell proliferation and cycle via PI3K/AKT signaling pathway (23–25). We found that overexpression of *SNHG12* significantly promoted the phosphorylation of AKT, while *SNHG12* knockdown inhibited the phosphorylation of AKT. It has been shown that the active Akt induced by PI3K could regulate the expression of its target genes, such as Bad, caspase-9, NF-κB, mTOR and p21, participating in the cell proliferation and cell apoptosis (26). Therefore, *SNHG12* may promote colorectal cell proliferation and cycle by activating PI3K/AKT signaling pathway. However, the underlying mechanism needs to be further investigated.

In conclusion, our data indicated that *SNHG12* might be a valuable diagnostic and prognostic biomarker for CRC and a potential target for gene therapy.
